# A tryptophan-rich peptide acts as a transcription activation domain

**DOI:** 10.1186/1471-2199-11-85

**Published:** 2010-11-16

**Authors:** Chen-Huan Lin, Grace Lin, Chia-Pei Chang, Chien-Chia Wang

**Affiliations:** 1 Department of Life Science, National Central University, Jung-li 32001, Taiwan

## Abstract

**Background:**

Eukaryotic transcription activators normally consist of a sequence-specific DNA-binding domain (DBD) and a transcription activation domain (AD). While many sequence patterns and motifs have been defined for DBDs, ADs do not share easily recognizable motifs or structures.

**Results:**

We report herein that the N-terminal domain of yeast valyl-tRNA synthetase can function as an AD when fused to a DNA-binding protein, LexA, and turn on reporter genes with distinct LexA-responsive promoters. The transcriptional activity was mainly attributed to a five-residue peptide, WYDWW, near the C-terminus of the N domain. Remarkably, the pentapeptide *per se *retained much of the transcriptional activity. Mutations which substituted tryptophan residues for both of the non-tryptophan residues in the pentapeptide (resulting in W_5_) significantly enhanced its activity (~1.8-fold), while mutations which substituted aromatic residues with alanine residues severely impaired its activity. Accordingly, a much more active peptide, pentatryptophan (W_7_), was produced, which elicited ~3-fold higher activity than that of the native pentapeptide and the N domain. Further study indicated that W_7 _mediates transcription activation through interacting with the general transcription factor, TFIIB.

**Conclusions:**

Since W_7 _shares no sequence homology or features with any known transcription activators, it may represent a novel class of AD.

## Background

Eukaryotic transcriptional activators that stimulate transcription initiation of a particular set of target genes usually consist of a sequence-specific DNA-binding domain (DBD) and a transcription activation domain (AD). The DBD targets these activators to a specific location in the promoter region of a gene, and the AD mediates transcription initiation by recruiting gene-specific factors, chromatin-remodeling factors, mediator complexes, and general transcription factors [1,2]. DBDs are classified into several distinct patterns or motifs according to their sequences and structural similarities. In contrast, ADs do not share significant sequence homologies, and therefore no specific motif has been defined. Despite this, several classes of ADs with distinctive sequence features were identified, including acidic activators [3], glutamine-rich activators [4], and proline-rich activators [5]. While an increasing number of transcriptional activators have been identified, the direct targets or interacting partners of most transcriptional activators and the detailed mechanisms through which these interacting partners induce transcription initiation remain largely unknown. To date, only a few protein factors have been explicitly identified as direct targets of transcriptional activators, including the TATA box-binding protein (TBP), TFIIB, TFIIH, and a few others [6].

One of the earliest and best-studied models for transcriptional regulation in eukaryotes is that of yeast Gal4, which is involved in regulating galactose metabolism in response to changes in the concentration of the carbohydrate [2,7]. The Gal4 system (also known as the *GAL *regulon) consists of four structural (*GAL1*, *GAL2*, *GAL7*, and *GAL10*) and three regulatory (*GAL3*, *GAL4*, and *GAL80*) genes. Protein products of the structural genes are required for transport and metabolism of galactose, and protein products of the regulatory genes control expression of the structural genes. Gal4 is an acidic transcriptional activator comprised of two functionally independent domains: an N-terminal sequence-specific DBD and a C-terminal AD. Induction of the Gal4-responsive genes by galactose is mediated through the specific binding of the Gal4-DBD to an upstream activating sequence in their promoter regions and subsequent recruitment of the general transcription apparatus by the Gal4-AD. Under non-inducing conditions in the absence of galactose, Gal4 activity is repressed by the interaction of Gal80 with the Gal4-AD [8,9]. Under inducing conditions when galactose is present, the repressor, Gal80, is taken away by Gal3 from the Gal4-AD, which is then able to recruit the transcription machinery and initiate transcription [10].

In prokaryotes, there are typically 20 aminoacyl-tRNA synthetases, one for each amino acid [11-14]. In eukaryotes, protein synthesis occurs in the cytoplasm, and also in organelles, such as mitochondria and chloroplasts [15]. Thus, eukaryotes, such as yeast, commonly have two genes that encode distinct sets of proteins for each aminoacylation activity, one localized in the cytoplasm and the other in the mitochondria. However, in some cases, the cytoplasmic and mitochondrial forms of a tRNA synthetase with a given amino acid specificity are encoded by the same nuclear gene through alternative initiation of translation, examples of which include *ALA1 *(which codes for alanyl-tRNA synthetase) [16], *GRS1 *(which codes for glycyl-tRNA synthetase) [17], *HTS1 *(which codes for histidyl-tRNA synthetase) [18], and *VAS1 *(which codes for valyl-tRNA synthetase (ValRS)) [19]. Because the two isoforms are essentially generated from the same open reading frame, they have the same polypeptide sequence, except for a short sequence attached to the amino-terminus of the mitochondrial precursor that functions as a mitochondrial targeting signal. As a consequence, the two isoforms cannot be substituted for each other *in vivo*.

Many eukaryotic cytoplasmic tRNA synthetases contain an amino- or carboxyl-terminal polypeptide extension, which is absent from their bacterial counterparts [20]. These extensions are generally rich in lysine residues and capable of non-specifically binding to RNA. A well-studied example is the appended domain (Ad) of yeast glutaminyl-tRNA synthetase (GlnRS), which binds unfractionated yeast tRNAs, single-stranded RNA, and pseudoknot RNA with comparable affinities, with *K*_d _values of around 0.6 μM [21,22]. Many other examples were also identified, such as the carboxyl-terminal domain of rice methionyl-tRNA synthetase [23] and the amino-terminal domain of mammalian lysyl-tRNA synthetase [24]. In addition to RNA binding, the Ads of some tRNA synthetases were found to participate in protein-protein interactions, such as those of yeast glutamyl- and methionyl-tRNA synthetases (GluRS and MetRS) [25] and mammalian ValRS [26]. However, more generally, the exact role of the Ad in the biological functions of this family of enzymes remains elusive. Recently, it was found that many of these Ads contain one or several classical nuclear localization signals [27] which are believed to play a role in the nuclear importation of these otherwise "cytoplasmic" proteins. Even more exciting are the findings that several tRNA synthetases from both prokaryotes and eukaryotes take part in functions unrelated to aminoacylation, including roles in mitochondrial RNA splicing, transcriptional and translational regulation, cytokine-like activity, and amino acid biosynthesis [28,29].

As with many known yeast tRNA synthetases, the cytoplasmic form of yeast ValRS also contains an amino-terminal polypeptide extension. While the Ad of mammalian ValRS was shown to interact with the four subunits of the elongation factor, EF-1H, to form a high-molecular-weight complex [26], relatively little is known about the biological function of its yeast counterpart. Our earlier studies suggested that the Ad of yeast ValRS (residues 1~98) possesses non-specific tRNA-binding activity (with a *K*_d _of ~2 μM) that significantly contributes to tRNA binding and aminoacylation activities of the enzyme [30,31]. We report herein that the N-terminal domain (residues 1~135) of yeast ValRS can act as an AD when fused to a sequence-specific DBD, and this transcriptional activity is mainly attributable to a tryptophan-rich peptide (WYDWW) within the N domain. Using this pentapeptide as a reference structure, a much more active peptide, heptatryptophan (W_7_), was consequently devised. Furthermore, W_7 _stimulated transcription initiation via interaction with TFIIB, a general transcription factor. It is our hope that information obtained in this study will advance our understanding of the biochemical properties of ADs in general, and also provide new insights into the mechanisms of transcription activation in particular.

## Results

### Identification of an activation peptide

**Figure 1:**
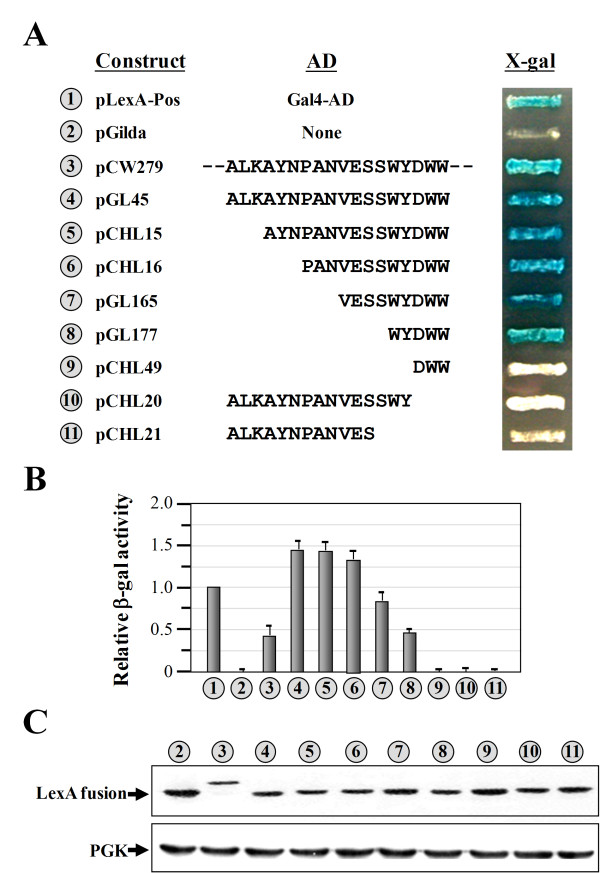
**Assays of the transcriptional activity of the N domain of yeast valyl-tRNA synthetase**. (**A**) Transcriptional assays. Various segments of the N domain of yeast ValRS were expressed as fusions to the specific DNA-binding protein, LexA, and the abilities of the resulting fusions to turn on the reporter genes (*LEU2 *and *lacZ*) which are controlled by distinct LexA-responsive promoters were tested. Activation of the reporter genes was indicated by the ability of the transformants to grow on selection medium lacking histidine, uracil, and leucine and to turn blue. (**B**) Quantitative assays of β-gal activity. (**C**) Western blot analysis of the expressions of LexA fusion proteins. *Upper panel*, LexA fusion protein; *lower panel*, phosphoglycerate kinase (PGK) (as a loading control). The numbers (circled) in **A**, **B**, and **C **represent the constructs shown in **A**.

In addition to tRNA binding, we wondered whether the N-terminal domain of yeast ValRS possesses another function. To explore this possibility, the N-terminal domain (residues 1~135) was cloned by fusion to the DNA-binding protein, LexA, and used as bait to screen a yeast library for interacting partners. Note that the N-terminal sequence used in this assay was 37 residues longer than the Ad (residues 1~98) used in previous studies [30,31] to ensure that all of the sequence elements that might be important for interactions were included. After all, the exact length of the Ad has yet to be clearly defined. The DNA sequence encoding the N-terminal domain was PCR-amplified and cloned as a *lexA *fusion into pGilda (carrying an *HIS3 *marker) as described in "Materials and Methods", and the resulting construct was cotransformed with the reporter plasmid, p8oplacZ (carrying a *URA3 *marker), into the EGY48 yeast strain. To rule out the possibility that the bait hybrid protein was itself an autoactivator, the cotransformants were first tested on selection medium (minimal medium containing X-gal but lacking uracil, histidine, and leucine) in the absence of any prey hybrid protein. Unexpectedly, the LexA-N domain fusion by itself could turn on the *LEU2 *and *lacZ *reporter genes; the cotransformants grew robustly on the X-gal agar plate and turned blue (Figure 1A, *number 3*). This result suggests that the bait hybrid protein *per se *is a transcription activator, and the N domain of yeast ValRS acts as an AD.

It was noted that the operator sequences in the reporter genes *LEU2 *and *lacZ *were not identical, and only fusions that could turn on both reporter genes were considered positive in this assay. To determine which segment of the N domain actually accounted for this transcriptional activity, various segments of the domain were individually fused to LexA, and their transcriptional activities were tested. As shown in Figure 1A, segments containing residues 98~115, 101~115, 104~115, 107~115, and 111~115 were active in the transcriptional assay (*numbers 4*~*8*). In contrast, segments consisting of residues 113~115, 98~112, and 98~109 had no detectable activity (*numbers 9*~*11*). Thus, the segment containing residues 111~115 (WYDWW) is essential and sufficient for this activity (*number 8*).

Quantitative assays of the β-gal activity (encoded by the reporter gene carried on p8oplacZ) further showed that among these active peptides, the segments containing residues 98~115, 101~115, and 104~115 had the highest activities (~1.5-fold relative to that of the positive control); the segment containing residues 107~115 had medium activity (~0.8-fold relative to that of the positive control); and the segments containing residues 1~135 and 111~115 had the lowest activities (~0.5-fold relative to that of the positive control) (Figure 1B). As expected, functionally inactive peptides had no detectable β-gal activity in the assays (Figure 1B, *numbers 9~11*). To check whether all of these LexA fusion constructs were properly expressed in the reporter yeast strain, the expression profiles of these constructs were analyzed by Western blotting using an anti-LexA antibody. As shown in Figure 1C, all of the LexA fusion proteins were stably expressed by the constructs, with only minor variations in protein levels, suggesting that the negative phenotype observed on the X-gal plate for the functionally inactive peptides was not caused by severe protein degradation or insufficient protein synthesis (Figure 1A, *numbers 9~11*). pLexA-Pos (Gal4 fused to LexA) and pGilda respectively served as positive and negative controls in the assays.

### Repetition of the pentapeptide sequence enhanced activity

**Figure 2:**
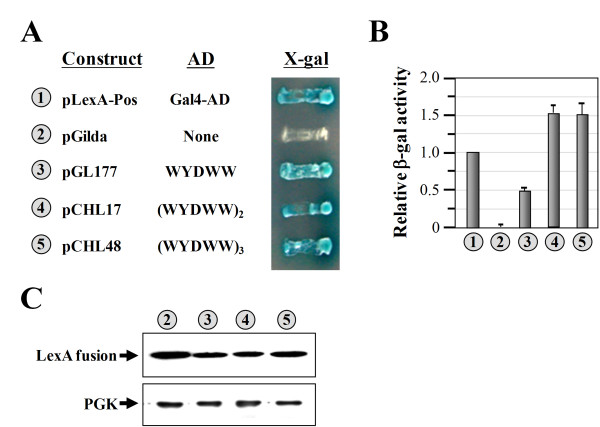
**Assays of the transcriptional activities of the pentapeptide and its tandem repeats**. (**A**) Transcriptional assays. The pentapeptide, WYDWW, and its tandem repeats were expressed as fusions to the specific DNA-binding protein, LexA, and the abilities of the resulting fusion proteins to turn on the reporter genes (*LEU2 *and *lacZ*), which are controlled by distinct LexA-responsive promoters, were tested. (**B**) Quantitative assays of β-gal activity. (**C**) Western blot analysis of the expressions of LexA fusion proteins. *Upper panel*, LexA fusion protein; *lower panel*, PGK (as a loading control). The numbers (circled) in **A**, **B**, and **C **represent the constructs shown in **A**.

While the pentapeptide, WYDWW, can function as an AD, its activity was relatively low, only ~50% of that of the positive control and ~30% of that of the segment containing residues 98~115 of the N domain (Figure 1B, *numbers 1*, *4*, and *8*). To enhance its activity, two or three tandem repeats of the pentapeptide sequence were cloned, and the activities of the resulting constructs were tested. As shown in Figure 2A and 2B, duplication of the pentapeptide, resulting in (WYDWW)_2_, strongly enhanced the activity (~3-fold increase relative to that of a single pentapeptide) (compare *numbers 3 *and *4*). However, an additional replication of the sequence, resulting in (WYDWW)_3_, did not further enhance the activity; (WYDWW)_3 _exhibited activity comparable to that of (WYDWW)_2 _(compare *numbers 4 *and *5*). Western blotting assays showed that these constructs expressed similar levels of LexA fusion proteins. Thus, changes in the transcriptional activity of these fusion constructs did not result from different protein expression levels (Figure 2C, *numbers 3~5*).

### W_5 _had activity higher than that of WYDWW

**Figure 3:**
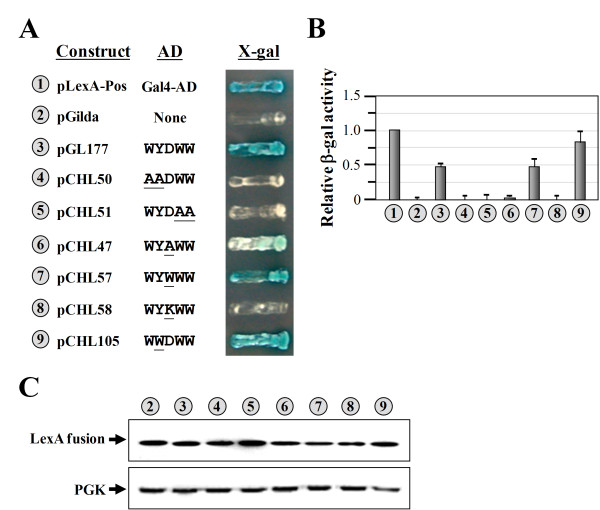
**Assays of the transcriptional activities of native and mutant pentapeptides**. (**A**) Transcriptional assays. Native and mutant pentapeptides were expressed as fusions to the specific DNA-binding protein, LexA, and the abilities of the resulting fusion proteins to turn on the reporter genes (*LEU2 *and *lacZ*), which are controlled by distinct LexA-responsive promoters, were tested. (**B**) Quantitative assays of β-gal activity. (**C**) Western blot analysis of the expressions of LexA fusion proteins. *Upper panel*, LexA fusion protein; *lower panel*, phosphoglycerate kinase (PGK) (as a loading control). The numbers (circled) in **A**, **B**, and **C **represent the constructs shown in **A**.

To examine which amino acid residues of the pentapeptide are critical for its activity, the residues were mutated to alanine residues, and the activities of the resulting constructs were tested. As shown in Figure 3, mutation of the first two amino acid residues (WY) to AA, the last two residues (WW) to AA, or the middle residue (D) to A or K drastically reduced the activities (compare *numbers 3*, *4*, *5*, *6*, and *8*). In contrast, mutation of the middle residue (D) to W had little effect on the activity (compare *numbers 3 *and *7*). Moreover, mutation of the second residue (Y) to W significantly enhanced the activity (~1.8-fold) (compare *numbers 3 *and *9*). Thus, it appears that W is preferred in all positions of the pentapeptide for activity. A Western blot analysis showed that these mutations had only minor effects on the expression levels of the fusion proteins (Figure 3C).

**Figure 4:**
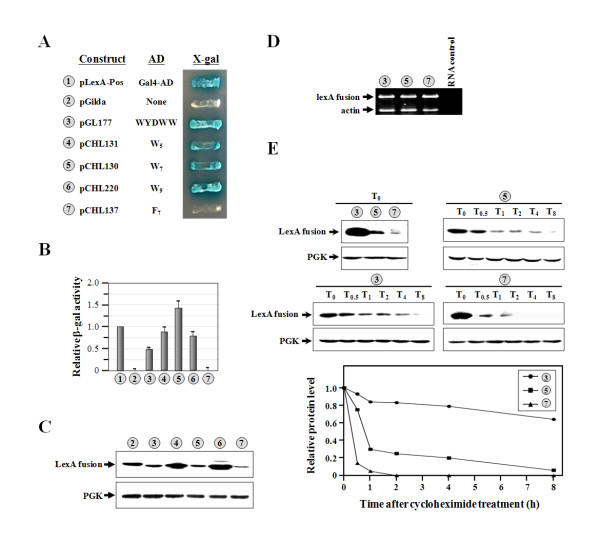
**Assays of the transcriptional activities of W**_**5**_**, W**_**7**_**, and W**_**9**_. (**A**) Transcriptional assays. W_5_, W_7_, and W_9 _were expressed as fusions to the specific DNA-binding protein, LexA, and the abilities of the resulting fusion proteins to turn on the reporter genes (*LEU2 *and *lacZ*), which are controlled by distinct LexA-responsive promoters, were tested. (**B**) Quantitative assays of β-gal activity. (**C**) Western blot analysis of the expressions of LexA fusion proteins. (**D**) RT-PCR. Relative levels of specific *lexA *mRNAs generated from each construct were determined by RT-PCR (28 cycles). Actin served as an internal control. "*RNA control*" denotes no reverse transcription for RNA prepared from W_7_. (**E**) Degradation assay. These constructs were expressed under the control of an inducible *GAL1 *promoter. *Upper panel*, LexA fusion protein; *lower panel*, PGK (as a loading control). *T*_*0*_, *T*_*0.5*_, *T*_*1*_, *T*_*2*_, *T*_*4*_, and *T*_*8 *_mean 0, 0.5, 1, 2, 4, and 8 h post-induction, respectively. The numbers (circled) in **A **to **E **represent the constructs shown in **A**.

To gain further insights, both of the non-W residues in the pentapeptide were mutated to W residues (resulting in W_5_), and the activity of the resulting construct was tested. As expected, W_5 _had an activity ~1.8-fold higher than that of the native pentapeptide (Figure 4A, B, *numbers 3 *and *4*). Most amazingly, inserting two more W residues into W_5_, yielding W_7_, further enhanced the activity (~1.8-fold) (compare *numbers 4 *and *5*). That is, the activity of W_7 _was ~3-fold higher than that of the native pentapeptide (compare *number 3 *and *5*). However, the activity of W_9 _was almost equivalent to that of W_5 _(*numbers 4 *and *6*). Thus, W_7 _appeared to be the strongest AD among those tested. To investigate whether the transcriptional activities of these peptides were attributable to their hydrophobic property, W_7 _was mutated to F_7_, and the activity of the resulting construct was tested. As shown in Figure 4A and 4B, changing W_7 _to F_7 _resulted in a functionally inactive peptide that failed to turn on the reporter genes (*numbers 5 *and *7*). With respect to protein expression, these fusion constructs expressed considerably different levels of proteins. LexA-W_5 _and LexA-W_9 _produced the highest protein expression levels (Figure 4C, *numbers 4 *and *6*); LexA-WYDWW and LexA-W_7 _had medium protein expression levels (*numbers 3 *and *5*); and LexA-F_7 _had the lowest protein expression level (*number 7*).

To further investigate whether the different protein expression levels observed herein were caused by different protein stabilities *in vivo*, a cycloheximide-chase assay (or degradation assay) was carried out on the representative constructs, LexA-WYDWW, LexA-W_7_, and LexA-F_7_. To exclude the interference of transcription activation, the host cell used for the assay was INVSc1, instead of the reporter yeast strain, EGY48. As shown in Figure 4E, these fusion proteins had conspicuously distinct turnover rates. LexA-WYDWW was much more stable than LexA-W_7_, and LexA-W_7 _was much more stable than LexA-F_7_. LexA-F_7 _had a short half-life (of <15 min) and was degraded *in vivo *at a much faster speed than the other two fusion proteins tested. It remains to be seen whether this attribute actually accounted for the negative phenotype of LexA-F_7 _in the transcriptional assay (Figure 4A, B). Another interesting finding was the discovery that while LexA-W_7 _had the highest transcriptional activity among these three constructs, it did not have the highest protein stability or expression level (Figure 4, *numbers 3, 5*, and *7*). Thus, it appears that there were no direct correlations between transcriptional activity and protein expression levels in these instances. Figure 4D shows that similar levels of cDNA products were generated from these fusion constructs, suggesting that the sequences encoding WYDWW, W_7_, and F_7 _did not compromise the stability of the specific *lexA *mRNAs *in vivo*.

### W_7 _is a non-promoter-specific AD

**Figure 5:**
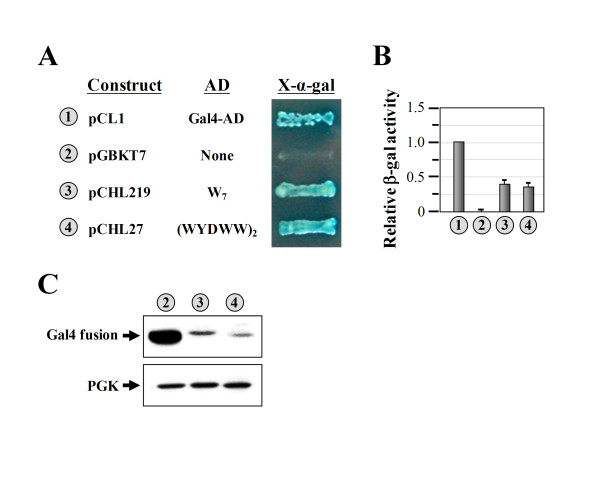
**Assays of the transcriptional activity of W**_**7 **_**using the Gal4-based system**. (**A**) Transcriptional assays. W_7 _and (WYDWW)_2 _were expressed as fusions to the Gal4-DBD, and the abilities of the resulting fusion proteins to turn on the reporter genes (*HIS3 *and *MEL1*), which are controlled by distinct Gal4-responsive promoters, were tested. (**B**) Quantitative assays of β-gal activity. (**C**) Western blot analysis of the expressions of Gal4-DBD fusion proteins. *Upper panel*, LexA fusion protein; *lower panel*, PGK (as a loading control). The numbers (circled) in **A**, **B**, and **C **denote the constructs shown in **A**.

We next tested whether the transcriptional activities of W_7 _and (WYDWW)_2 _were promoter-specific, and whether they were affected by the DBD used. To this end, W_7 _and (WYDWW)_2 _were assayed in a Gal4-based system, where the AD was fused in-frame to the Gal4-DBD cloned in pGBKT7 (which carries a *TRP1 *marker), and the reporter genes used were *HIS3 *and *MEL1 *(which encodes α-galactosidase) under the control of two completely heterologous Gal4-responsive upstream activating sequences and promoter elements, *GAL1 *and *MEL1*, respectively. As shown in Figure 5, both Gal4-DBD fusion proteins turned on the reporter genes; transformants carrying either of these two fusion constructs (Gal4-DBD-W_7 _or Gal4-DBD-(WYDWW)_2_) grew robustly and turned blue on selection medium containing X-α-gal but lacking tryptophan and histidine (Figure 5A, *numbers 3 *and *4*), suggesting that both of these peptides acted as ADs in Gal4-DBD fusion proteins. Thus, the transcriptional activities of these two peptides were non-promoter-specific and were operational in both LexA- and Gal4-DBD-responsive reporter genes.

Quantitatively, these two fusion proteins had transcriptional activities ~2.5-fold lower than that of the positive control (Figure 5B, *numbers 1*, *3*, and *4*). This is not surprising, considering the fact that the positive control was a wild-type *GAL4 *gene. Western blot assays showed that these two Gal4-DBD fusion constructs had protein expression levels much lower than that of the Gal4-DBD alone (Figure 5C, *numbers 2*~*4*). Whether the ADs destabilized the Gal4-DBD fusion proteins and whether the relatively poor transcriptional activity of these two fusion constructss was caused by a lower level of protein expression are yet to be determined. However, regardless of the diverse protein expression levels, these results clearly demonstrate the ability of these two peptides to function as ADs in the Gal4-based system.

### W_7 _acts as an AD in a two-hybrid system

**Figure 6:**
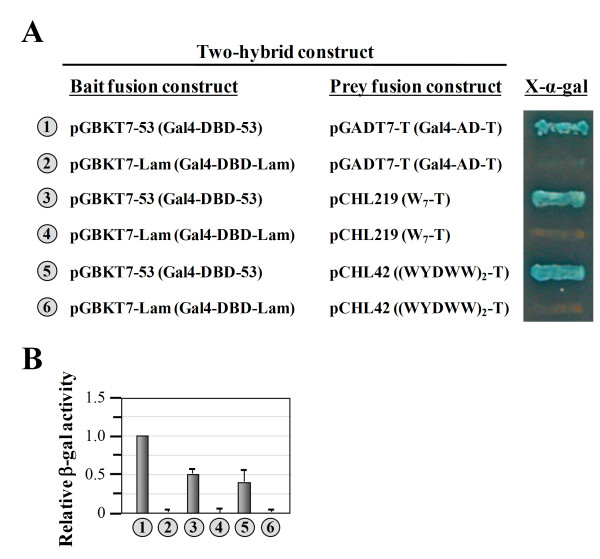
**Assays of the transcriptional activity of W**_**7 **_**using a two-hybrid system**. (**A**) Two-hybrid assays. W_7 _and (WYDWW)_2 _were expressed as fusions to the SV40 large T-antigen, and the abilities of the resulting fusions to specifically interact with Gal4-DBD-53 and turn on the reporter genes (*HIS3 *and *MEL1*) were tested. For clarity, the bait and prey fusion proteins encoded by the constructs are depicted in parentheses following the vector names. (**B**) Quantitative assays of β-gal activity. The numbers (circled) in **A **and **B **represent the constructs shown in **A**.

So far, we have shown that W_7 _and (WYDWW)_2 _can act as ADs when directly fused to a sequence-specific DBD such as LexA and Gal4-DBD (Figures 2, 4, 5). The question arose as to whether these two short peptides can act as efficiently when they are physically separated from the DBD. To answer this question, DNA sequences encoding these two peptides were individually cloned into pGADT7-T (a prey fusion vector encoding the Gal4-AD-T fusion protein) to replace the sequence encoding the Gal4-AD, and the transcriptional activities of the resulting constructs were tested following the protocols devised for the yeast two-hybrid system. As shown in Figure 6, murine p53 (as a part of the Gal4-DBD-53 fusion) specifically interacted with the SV40 large T-antigen (as a part of the Gal4-AD-T fusion) and turned on the reporter genes, *HIS3 *and *MEL1 *(*number 1*, serving as the positive control). In contrast, human lamin C (as a part of the Gal4-DBD-Lam fusion) did not interact with the large T-antigen and therefore failed to turn on the reporter genes (*number 2*, serving as the negative control). Interestingly, both W_7_-T and (WYDWW)_2_-T fusions turned on the reporter genes when acting in concert with the Gal4-DBD-53 fusion (*numbers 3 *and *5*), but failed to do so when acting in concert with the Gal4-DBD-Lam fusion (*numbers 4 *and *6*). This observation provides strong evidence that both W_7 _and (WYDWW)_2 _can act as ADs in a traditional two-hybrid system, albeit with efficiencies poorer than that of Gal4-AD (Figure 6B, *numbers 1*, *3*, and *5*).

### W_7 _mediates transcription activation through interacting with TFIIB

**Figure 7:**
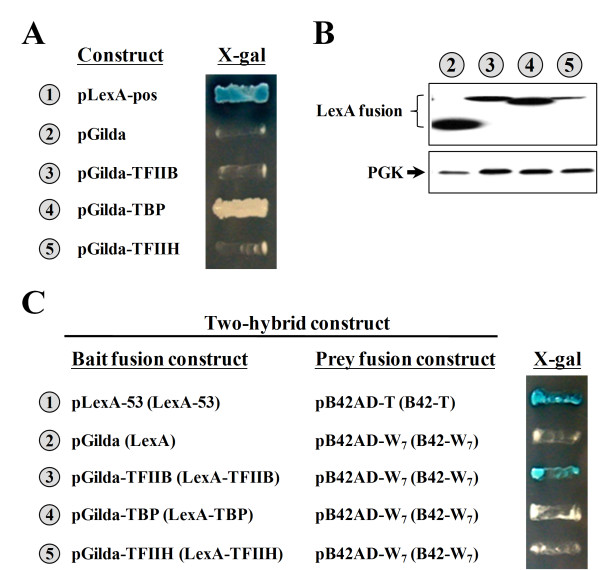
**Identification of the interacting partners of W**_**7 **_**using a two-hybrid system**. (**A**) Transcriptional assay. TBP, TFIIB, and TFIIH were expressed as fusions to LexA, and the abilities of the resulting fusions to turn on the reporter genes (*LEU2 *and *lacZ*) were tested. (**B**) Western blot analysis of the expressions of LexA fusion proteins. *Upper panel*, LexA fusion protein; *lower panel*, PGK (as a loading control). The numbers (circled) in **A **and **B **denote the constructs shown in **A**. (**C**) Two-hybrid assays. TBP, TFIIB, and TFIIH were expressed as fusions to LexA, and the abilities of the resulting fusions to specifically interact with W_7 _and turn on the reporter genes (*LEU2 *and *lacZ*) were tested. For clarity, the bait and prey fusion proteins encoded by the constructs are depicted in parentheses following the vector names.

To date, only a few protein factors have unambiguously been identified as direct targets of transcriptional activators, including TBP, TFIIB, TFIIH, and a few others [6]. We wondered whether W_7 _also stimulates transcription initiation through interaction with one of these protein factors. Pursuant to this objective, TBP, TFIIB, and TFIIH were individually cloned into pGilda (a bait fusion vector encoding the LexA fusion protein), and then their interaction with W_7 _(as a part of the B42-W_7 _fusion protein) was tested using a yeast two-hybrid system. LexA and B42 respectively served as the DBD and AD in this assay. As shown in Figure 7, all of these LexA fusion proteins were well expressed in the test yeast strain EGY48 (Figure 7B), with LexA-TFIIH having a lower expression level. In the absence of W_7_, none of these fusion constructs *per se *could simultaneously turn on the designated reporter genes, *LEU2 *and *lacZ *(Figure 7A). However, in the presence of W_7_, TFIIB, but not TBP or TFIIH, could turn on both reporter genes (Figure 7C). This result strongly suggests that W_7 _specifically interacts with TFIIB.

## Discussion

We report herein that the N-terminal domain of yeast ValRS acts as an AD when fused to a sequence-specific DBD, LexA (Figure 1). However, there is no direct evidence so far showing that yeast ValRS actually functions as a transcription activator in the nucleus. More experiments are currently underway to validate this possibility. Despite this uncertain aspect, a core sequence element accounting for this activity was mapped to a region near the C-terminus of the N domain. This core sequence element contains only five amino acid residues: WYDWW (N-terminal residues 111~115) (Figure 1). Interestingly, the core sequence element *per se *retained much of the transcriptional activity and could be stably expressed as a fusion to the DBD in yeast (Figure 1). A mutagenesis study indicated that W residues in the pentapeptide were crucial for its activity. Even the Y residue, which also possesses an aromatic ring in its side chain, was not as supportive of the activity as were the W residues (Figure 3). As a result, the native pentapeptide acted less efficiently (~1.8-fold) than did WWDWW and WWWWW as an AD (Figures 3, 4). Using this core sequence element as a reference, a much-stronger activation peptide, W_7_, was successfully produced. W_7 _had ~3-fold higher transcriptional activity than those of the native pentapeptide and the N domain (Figures 1, 4). While we cannot rule out the possibility that W_7 _somehow altered the affinity or specificity for the *lexA *operators of the LexA fusion protein, W_7 _has to be capable of interacting with one of the components of the transcription apparatus to stimulate transcription of the reporter genes. To our knowledge, W_7 _is so far the smallest AD that retains strong transcriptional activity. With its small size, W_7 _is unlikely to interfere with the folding of fused proteins or to occlude the normal site of interaction. In addition, W_7 _can significantly reduce the overall size of the prey fusion construct, and therefore has the potential to enhance the efficiency of plasmid transformation, which is a long-standing issue when screening a large library for novel interacting partners. Similar to the reasons that the His_6 _tag is widely accepted and used for protein purification, W_7 _may be an excellent replacement for the Gal4-AD or B42 in two-hybrid screening as an AD.

A preliminary study suggested that the direct target of W_7 _may be the general transcription factor, TFIIB (Figure 7), which was previously shown to be one of the interacting partners of the acidic transcription activator, Gal4 [10]. It is believed that recruitment of TFIIB and others by Gal4 leads to assembly of the transcription apparatus at the promoter site and subsequent transcription initiation [7]. However, sequence alignment between these two ADs (Gal4-AD and W_7_) showed little sequence homology. Thus, Gal4-AD and W_7 _may interact with different sites of TFIIB or through a different mechanism. Alternatively, a certain portion of Gal4 may fold into a three-dimensional structure that displays a hydrophobic feature similar to that of W_7_. In any case, it is interesting to find that both of these ADs mediate transcription activation through interacting with TFIIB. Thus, the minute AD, W_7_, might turn out to be an interesting paradigm for further mechanistic studies of transcription activation.

## Conclusions

A short peptide containing seven consecutive tryptophan residues (W_7_) can function as an activation domain when fused to a DNA-binding protein, LexA, and turn on reporter genes with distinct LexA-responsive promoters. Like the activation domain of Gal4, W_7 _mediates transcription activation through interacting with the general transcription factor, TFIIB.

## Methods

### Construction of various LexA and Gal4-DBD fusion constructs

To clone the DNA sequence encoding the N domain of yeast ValRS (base pairs +1 to +405) into pGilda and pGBKT7 (BD Biosciences Clontech, Palo Alto, CA) for the transcriptional assays, a set of primers complementary to nucleotides -15 to +15 and +390 to +420 of *VAS1 *were respectively used to amplify the DNA fragment using a plasmid-borne *VAS1 *gene as a template. The forward primer contained an EcoRI site, and the reverse primer contained an XhoI site. The 405-bp PCR fragment was first digested with EcoRI and XhoI and then cloned into appropriate sites in pGilda or pGBKT7. To clone shorter fragments of the N domain, such as those coding for amino acid residues 98~115 and 111~115, or their derivatives, such as those coding for W_5_, W_7_, and W_9_, into these two vectors, a pair of complementary oligonucleotides coding for the desired sequences were annealed and then cloned into the EcoRI-XhoI sites of these vectors. Sequences of the cloned DNA fragments were verified by DNA sequencing.

### Transcriptional assay

This assay essentially followed the protocol of a yeast two-hybrid assay provided by the manufacturer (BD Biosciences Clontech), except that the prey cloned in pB42AD was not used. Briefly, the gene of interest was first cloned as a *lexA *fusion into pGilda (carrying an *HIS3 *marker) as mentioned above. The *lexA *fusion construct was then co-transformed with the reporter plasmid, p8oplacZ (carrying a *URA3 *marker and a *lacZ *reporter gene under the control of eight copies of the LexA operator and the minimal *GAL1 *promoter), into the EGY48 yeast strain (carrying an *LEU2 *reporter gene under the control of six copies of the LexA operator and the minimal *LEU2 *promoter), and the resulting cotransformants were selected on minimal medium lacking uracil and histidine. A single colony of the cotransformants that grew on the selection medium was picked and streaked on an agar plate containing X-gal but lacking uracil, histidine, and leucine. The streaked colony could not grow on the X-gal plate and turn blue unless the LexA fusion protein had turned on both reporter genes (*LEU2 *and *lacZ*). Note that the DNA-binding protein, LexA, alone could not activate transcription of the LexA-responsive reporter genes, but could do so when fused to an AD.

Alternatively, the transcriptional activity of the N domain was tested using a Gal4-based system (BD Biosciences Clontech), in which the DNA sequence encoding the N domain was fused in-frame to the sequence encoding Gal4-DBD cloned in pGBKT7 (carrying a *TRP1 *marker), and the resulting construct was then transformed into the AH109 reporter yeast strain and tested for its ability to turn on the reporter genes, *HIS3 *and *MEL1*. *HIS3 *was under the control of the *GAL1 *upstream activating sequence and a minimal promoter containing the *GAL1 *TATA box. The expression of *MEL1 *was controlled by the intact *MEL1 *promoter, including the *MEL1 *upstream activating sequence and *MEL1 *minimal promoter. The transformants could not grow and turn blue on selection medium containing X-α-gal but lacking tryptophan and histidine unless the Gal4-DBD fusion protein had turned on both reporter genes.

### Western blot analysis

Protein expression patterns of the LexA fusions were determined by a chemiluminescence-based Western blot analysis. The transformants used in the transcriptional assays were grown in selection medium lacking uracil and histidine. Total protein extracts were prepared from the transformants with a buffer containing 50 mM Tris (pH 7.4), 150 mM NaCl, 0.5% sodium dodecylsulfate (SDS), 0.5% Triton X-100, 10 mM ethylenediaminetetraacetic acid, and 1 mM phenylmethanesulfonyl fluoride. Aliquots of the protein extracts (~40 μg) were loaded onto a mini gel (8 × 10 cm) containing 10% polyacrylamide and electrophoresed at 100 V for ~2 h. Following electrophoresis, the resolved proteins were transferred to a polyvinylidene difluoride (PVDF) membrane using a semi-dry transfer device. The membrane was probed with a horseradish peroxidase (HRP)-conjugated anti-LexA antibody (Invitrogen, Carlsbad, CA) and then exposed to x-ray film following the addition of appropriate substrates. The protein expression patterns of the Gal4-DBD fusions were determined following a similar protocol.

### β-Galactosidase (β-Gal) assay

Yeast cells were pelleted by centrifugation at 12,000 × *g *for 30 s and resuspended in 100 μl of breaking buffer (100 mM Tris-HCl (pH 8.0), 1 mM dithiothreitol, 10% glycerol, and 2 mM phenylmethanesulfonyl fluoride) and 100 μl of beads. Cells were then lysed at 4°C using a bead beater, followed by centrifugation at 12,000 × *g *for 2 min. Aliquots of the supernatants (25~250 μg) were diluted to 0.8 ml with Z buffer (60 mM Na_2_HPO_4_, 40 mM NaH_2_PO_4_, 10 mM KCl, 1 mM MgSO_4_, and 50 mM 2-mercaptoethanol). β-Gal activity assays were initiated (at 37°C) by adding 0.2 ml of o-nitrophenyl β-D-galactoside (4 mg/ml). The reaction mixtures were incubated with constant shaking at 37°C for 20 min and then terminated by the addition of 0.4 ml of 1 M Na_2_CO_3_. The reaction mixtures were centrifuged at 12,000 × *g *for 2 min, and the absorbance (*A*_420_) of the supernatants was determined. Relative β-gal activities were calculated from *A*_420 _readings normalized to protein concentrations. Data were obtained from three independent experiments and averaged. Error bars indicate (± 2 × standard deviation).

### Reverse-transcription (RT)-PCR

To determine the relative levels of specific *lexA *mRNAs derived from the fusion constructs, a semiquantitative RT-PCR experiment was carried out following the protocols provided by the manufacturer (Invitrogen). Total RNA was first isolated from the transformants and then treated with DNase to remove contaminating DNA. Aliquots of RNA (~1 μg) were then reverse-transcribed into single-stranded complementary (c)DNA using an oligo-dT primer. After RNase H treatment, the single-stranded cDNA products were amplified by a PCR using a pair of specific primers. The forward and reverse primers respectively contained sequences complementary to nucleotides +1 to +21 (5'-ATGAAAGCGTTAACGGCCAGG-3') and nucleotides +370 to +390 of *lexA *(5'-CAAGTCACCATCCATAATGCC-3'). As a control, the relative levels of actin-specific mRNAs in each preparation were also determined using a set of primers complementary to nucleotides +537 to +560 (5'-ACCAACTGGGACGATATGGAAAAG-3') and nucleotides +696 to +719 (5'-TTGGATGGAAACGTAGAAGGCTGG-3') of actin, respectively.

### Degradation assay

To determine the turnover of the fusion proteins, analogous *lexA *fusion constructs that were expressed under the control of an inducible *GAL1 *promoter were transformed into INVSc1. Transformants carrying these constructs were first grown in medium lacking histidine with 2% raffinose to a cell density of ~1.0 *A*_600 _and then induced with 2% galactose for 1 h. Afterward, cells were washed twice and then grown in medium containing 2% glucose and 100 μg/ml cycloheximide but lacking histidine. Cells were harvested at 0, 0.5, 1, 2, 4, and 8 h postinduction and lysed. Forty-microgram samples of the cellular lysates were resolved on 10% polyacrylamide and electrophoresed at 100 V for ~1 h, and the proteins were transferred to a PVDF membrane and immunoblotted with an HRP-conjugated anti-LexA antibody.

## Authors' contributions

CHL generated the various *lexA *constructs and performed the RT-PCR, Western blotting, and transcriptional assays. GL and CPC performed the degradation and β-galactosidase assays. CCW coordinated the project and wrote the manuscript. All authors read and approved the final manuscript.
